# Results from 10 Years of a CBT Pain Self-Management Outpatient Program for Complex Chronic Conditions

**DOI:** 10.1155/2016/4678083

**Published:** 2016-11-06

**Authors:** Kathryn A. Boschen, Edward Robinson, Kent A. Campbell, Sarah Muir, Elvina Oey, Kristen Janes, Samantha R. Fashler, Joel Katz

**Affiliations:** ^1^Bridgepoint Health, Toronto, ON, Canada; ^2^University of Toronto, Toronto, ON, Canada; ^3^York University, Toronto, ON, Canada

## Abstract

*Background.* Traditional unimodal interventions may be insufficient for treating complex pain, as they do not address cognitive and behavioural contributors to pain. Cognitive Behavioural Therapy (CBT) and physical exercise (PE) are empirically supported treatments that can reduce pain and improve quality of life.* Objectives.* To examine the outcomes of a pain self-management outpatient program based on CBT and PE at a rehabilitation hospital in Toronto, Ontario.* Methods.* The pain management group (PMG) consisted of 20 sessions over 10 weeks. The intervention consisted of four components: education, cognitive behavioural skills, exercise, and self-management strategies. Outcome measures included the sensory, affective, and intensity of pain experience, depression, anxiety, pain disability, active and passive coping style, and general health functioning.* Results.* From 2002 to 2011, 36 PMGs were run. In total, 311 patients entered the program and 214 completed it. Paired* t*-tests showed significant pre- to posttreatment improvements in all outcomes measured. Patient outcomes did not differ according to the number or type of diagnoses. Both before and after treatment, women reported more active coping than men.* Discussion.* The PMGs improved pain self-management for patients with complex pain. Future research should use a randomized controlled design to better understand the outcomes of PMGs.

## 1. Introduction

Although chronic pain has been variously defined, it is commonly accepted to encompass pain that has no biological value, that persists beyond the usual healing and rehabilitation times, or that is not responsive to currently available treatments [1]. Individuals with chronic pain place substantial strain on the healthcare system and often experience functional disabilities that reduce their quality of life (QOL) [2]. Health-related QOL describes the physical, mental, social, psychological, and functional aspects of wellbeing from the patient's perspective [3]. Chronic pain afflicts 20–30% of the adult population in western countries [4, 5] and often accompanies other complex chronic conditions [6]. Notably, a recent survey by Kuluski et al. [6] conducted at the same centre at which the present research was conducted, Bridgepoint Health, found that pain was the most commonly reported illness symptom by inpatients in a continuing care/rehabilitation hospital.

Patients frequently report that pain interferes with their ability to attend social and family events, to participate in recreational activities, and to carry out daily tasks [7]. Chronic pain contributes substantially to sick days and loss of workplace productivity, making it the leading cause of disability in the working-age population [8]. Particularly for those who are older or retired or are younger but seriously impacted by long-term pain, high utilization of healthcare services is common, with 38% of patients who present to primary care physicians with chronic pain [9].

Chronic pain is challenging to treat because it is a condition that consists of biological, psychological, and social components. Since most pain management strategies more typically focus on biological interventions, such as pharmacology, nerve blocks, and surgery, they do not address all of the components of the pain experience. This is especially important as the secondary consequences of living with chronic pain, such as depression and anxiety, often prove to be most detrimental to health-related QOL [11]. Chronic pain yields feelings of emotional distress, helplessness, and loss of control [12, 13]. Due to the complex nature of chronic pain, unimodal interventions that solely target the physical component of pain are frequently not sufficient. However, programs using Cognitive Behavioural Therapy (CBT) have been shown to be the most efficacious approach to symptom management as they address both psychological and functional components of health [13]. While CBT is delivered in both individual and group formats, the latter is more common and more cost-effective.

CBT is predicated on the notion that to understand pain one must also consider the cognitive and behavioural factors that influence the pain experience [14, 15]. CBT applies psychological principles to change behaviours, thoughts, and feelings of individuals with chronic pain to help them experience less distress. CBT encourages patients to conceptualize pain as manageable, to move from a passive to active role in pain control, and to develop adaptive behavioural and cognitive responses to pain [16]. A literature review of cognitive behavioural treatments indicated that these programs can reduce pain, restore lost function, enhance health-related QOL, and decrease reliance on medical care compared to unimodal treatments based on the biomedical model [17]. Dysvik et al. [18] found that therapeutic dialogue was the most successful component of their CBT program as it enabled group members with similar problems to develop a sense of community. In other CBT programs, strategies such as relaxation have been successfully used to cope with pain [19]. Turk et al. [5] emphasize that no single treatment will be efficacious alone and that a combination of treatment modalities such as those used in CBT will yield the best results.

Research supports this suggestion regarding interventions combining CBT and physical exercise (PE) to optimize the overall health and QOL of individuals with chronic pain. While CBT targets the psychosocial components of chronic pain, PE targets the biological component by overcoming physical deconditioning [20]. Turk and Okifuji [21] found that fear and avoidance of physical activities are more strongly associated with disability and work loss than the biomedical variables of pain. Their study underlines the importance of modifying negative thoughts surrounding exercise and engaging individuals with chronic pain in PE. Presently, only two studies have examined the impact of combining CBT and PE interventions on chronic pain, both resulting in significant reductions in pain intensity and significant improvements in health-related QOL [14, 18]. However, further research is required to evaluate the intervention's effectiveness over an extended period. Not only is the research sparse examining the effectiveness of pain self-management programs using both CBT and PE, but also the comparability of chronic pain programs is limited due to differences in service content and intensity, approaches to evaluation of results, and follow-up periods used [22].

The present study contributes to the growing literature on the combination of CBT and PE interventions through an evidence-based outcome analysis of 10 years of data accumulated between 2002 and 2011 from a pain self-management outpatient program at Bridgepoint Health, a rehabilitation hospital in Toronto, Ontario, for individuals with complex chronic conditions. The study purpose was to assess the impact of the CBT and PE-based pain management group (PMG) program by empirically examining participants' key health-related quality of life outcomes using six well-known published and standardized norm-based instruments administered through the PMG. The program remained consistent over the 10-year period due to the continuous delivery of the PMG by a core set of clinicians described below.

## 2. Methods

The study used a one-group quantitative design to evaluate outcomes of a 10-week PMG program over 36 consecutive groups through an analysis of change in six dependent variables comparing participants' health-related self-perceptions from preprogram time to postprogram time. Data from a large number of participants were used to enhance the external validity of the study and to lay the groundwork for a future randomized, prospective waitlist-control study. The study protocol was approved by the Research Ethics Boards at the University of Toronto and at the Joint Bridgepoint-West Park-Toronto Central Community Care Access Centre. Written informed consent was obtained from participants prior to beginning the study.

### 2.1. Participants

Data from 311 patients, 243 (78.1%) females and 68 (21.9%) males aged 18–70 years who had chronic pain, were included in the PMG dataset used in this study. The gender split is typical of CBT-based pain programs, which are heavily weighted by females.

Each participant met the following inclusion criteria: (1) 18 years of age or older, (2) chronic pain for at least six months, (3) medical condition previously fully assessed and medical management optimized, (4) experiencing adverse effects of pain on daily life, and (5) being interested in developing self-management strategies to cope with pain. Participants were excluded if (1) they were medically or psychiatrically unstable (e.g., severe depression, active psychosis, current substance abuse, and personality disorder preventing ability to interact effectively in a group program), (2) comprehension of English was too limited to participate in group discussions, and/or (3) pain was related to ongoing effects of metastatic cancer.

### 2.2. Program Description and Staffing

The PMG was introduced at Bridgepoint in 2002 by Edward Robinson, M.D., based on a program developed at Chedoke-McMaster in the 1970s by Eldon Tunks, M.D. Dr. Robinson had previous experience with similar pain programs at two other Toronto hospitals from 1988 to 2009. All referrals are made by physicians (family doctors or specialists). If the referral meets admission criteria for the PMG, the patient completes a questionnaire and attends an appointment for initial assessment to determine the appropriateness of the patient for the program. The patient's medical history is reviewed, the purpose and structure of the program are described, and the patient's expectations for the program are discussed. If the patient meets the inclusion criteria and is interested in participating in the group, an individual assessment with the physiotherapist and registered nurse is completed.

The assessment provides the physiotherapist with sufficient knowledge of the patient's condition and limitations to better direct him/her regarding the appropriate type and amount of exercise to do in group exercise sessions. The nurse conducts a medical screen including review of current medications and risk factors that might require modifications for participation in the program, such as heart conditions, diabetes, bladder incontinence, and allergies.

Participants attend the PMG twice weekly for 10 weeks (see Table 7 for a detailed description of session content). The 20 sessions include a 2-hour discussion followed by group exercise (1/2–1 hour in duration). No drop-in participants were permitted (i.e., groups were closed) and a maximum of 12 patients are enrolled in each group. Participants are given a program manual with over 100 pages of notes and information about the different aspects of the program, as well as pencil-and-paper exercises to complete for some sessions. Notes from some of the group discussions are added to the manual periodically during the program.

The purpose of the exercise component is to encourage patients to be as active as possible within the limits of their pain condition and to learn to do so under supervision of a therapist skilled in working with chronic pain patients. In this way, they come to know what is safe for them to do and are able to avoid exercise-induced pain flare-ups. Different exercise modalities are introduced so that all patients are taught individually modified exercises that they can continue in the community on their own, either at home or by joining a community program.

The physiotherapist leads a class of Qi Gong once a week. Qi Gong is an ancient form of Chinese energy exercise. It is the practice of developing greater awareness and control for therapeutic and healing purposes. The exercises can be done while seated or standing and are modified for patients with pain and physical disabilities [23]. Patients are given a DVD on which the exercises are illustrated so they can continue to exercise at home after the program is finished. Once a week, patients have the option to attend the warm therapeutic pool for a gentle aerobic exercise class led by the physiotherapy assistant. For those who cannot attend the pool sessions, the physiotherapist teaches individualized exercises in a small group.

The 2-hour group sessions are co-led by a physician, a social worker, and an occupational therapist or registered nurse depending on the content discussed in the session. The psychotherapeutic approach used is CBT, which is structured, interactive, educational, time-limited, and problem-oriented. It teaches self-management skills to enable patients to cope more effectively with their pain. Using home practice to consolidate the skills learned is an important aspect of the program. The complex nature of pain is discussed. Patients are given basic information about the neurophysiology of the pain system (peripheral and central nervous systems) to understand that the pain experience can be self-modified or moderated through a variety of techniques.

The relationship between pain and stress is discussed, as well as the emotional aspects and effects of pain, especially depression, anxiety, and anger. Other topics include sleep and sleep problems, diet and nutrition, medications for pain, energy conservation, sexuality, and relationship and family issues. Coupled with information about each topic are simple strategies to deal with the problem more effectively.

The occupational therapist leads a discussion on energy conservation. The nurse addresses issues of diet and nutrition related to pain. A sex therapist discusses the issues of sexuality and pain. One or two former group members are invited to attend a session to share their experiences and to address questions about how to continue pain management after the group program ends. A family session is offered to allow family members an opportunity to learn about chronic pain if a majority of members in the group have someone who is able to attend. Patients are introduced to specific self-management strategies such as relaxation exercises, guided pain imagery, modifying negative thoughts, and goal setting. They are given relaxation CDs and are encouraged to practice daily to develop their skills. Many patients find relaxation to be very effective in reducing their level of pain and/or pain-related stress. Patients are also taught guided pain imagery to modify the pain experience. This involves first a description of their perception of the pain as precisely as possible. Then, they experiment with imagery to modify the pain image. Once they have chosen a helpful image that is associated with pain relief for them, they are encouraged to practice repeatedly bringing that image to mind. This approach can alter the pain experience significantly in some patients.

Cognitive therapy techniques are emphasized in the group. Patients are taught that thoughts are not the same as objective reality; it is possible to acknowledge negative thoughts but then look for alternative, more helpful thoughts. They learn that they do have some choice about what they focus on and that while some of the thoughts may be objectively true, others may be distorted. The concepts of cognitive therapy are introduced through examples from previous group members, and then participants are encouraged to share examples from their own experience for discussion. The group acts as a resource for ideas and suggestions of alternative thoughts. This technique can become a life skill and a way to cope more effectively with problems, including the challenge of living with chronic pain.

During the first week of the program, participants are encouraged to set personal goals for the 10 weeks. The goals are reviewed in weeks 5 and 10. The purpose of goal setting is to encourage each patient to focus on practical life changes that are short-term and realistic, recognizing that gaining control over chronic pain is often achieved through a series of small steps over time. Goal setting is one of the self-management tools that enable patients with chronic pain to regain control of their lives.

### 2.3. Materials

Six questionnaires were administered at the start and end of the program.

#### 2.3.1. Short-Form McGill Pain Questionnaire (SF-MPQ)

The SF-MPQ provides information on the sensory, affective, and overall intensity of the pain experience [24]. The 15-item instrument uses a four-point scale, 0 (none), 1 (mild), 2 (moderate), and 3 (severe), of symptom severity to measure the sensory (11 items) and affective (4 items) dimensions of pain. The SF-MPQ also has a 10 cm visual analogue scale (VAS) that patients use to rate their overall pain intensity. The correlations between the SF-MPQ and the full MPQ are consistently high, and the test-retest reliability ranges from 0.88 to 0.96 [25, 26].

#### 2.3.2. Beck Depression Inventory (BDI-II)

The BDI-II measures depression [27]. Each participant self-administered the 21-item questionnaire on the basis of the preceding two weeks, using a 4-point scale: 0 (least severe) to 3 (most severe). Clinical analysis of total scores used the following guidelines: 0–13 (minimal), 14–19 (mild), 20–28 (moderate), and 29–63 (severe). The BDI-II has strong internal consistency (Cronbach's *α* = 0.92), excellent test-retest reliability (0.93), and high construct validity [28].

#### 2.3.3. Beck Anxiety Inventory (BAI)

The BAI assesses clinical anxiety [29]. Each participant completed the 21-item multiple-choice questionnaire on the basis of the preceding week. The items on the BAI measure four symptoms of anxiety: subjective, neurophysiological, autonomic, and panic-related. Participants chose one of four possible responses: 0 (not at all), 1 (mild), 2 (moderate), and 3 (severe). Clinical interpretation of the total scores used the following guidelines: 0–7 (minimal), 8–15 (mild), 16–25 (moderate), and 26–63 (severe). The BAI has strong internal consistency (Cronbach's *α* = 0.92–0.94), good test-retest reliability (0.75), and high construct validity [29].

#### 2.3.4. Pain and Disability Index (PDI)

The PDI measures the degree to which chronic pain interferes with daily activities. This 7-item instrument has an 11-point Likert Scale that ranges from 0 (no disability) to 10 (total disruption in daily activities) [30]. The PDI has good internal consistency (Cronbach's *α* = 0.86), modest test-retest reliability, and high concurrent validity [30].

#### 2.3.5. Vanderbilt Pain Management Inventory (VPMI)

The VPMI was developed to assess the coping mechanisms used by chronic pain patients to manage moderate to severe pain episodes [31]. This 18-item measure uses a 5-point scale to indicate the frequency of each strategy used ranging from 1 (never do when in pain) to 5 (very frequently do when in pain). The VPMI has fair internal consistency (Cronbach's *α* = 0.71–0.82), high concurrent validity, and high predictive validity [31].

#### 2.3.6. Short-Form Health Survey (SF-36)

The SF-36 is a measure of general health that consists of eight scaled scores: vitality, physical functioning, bodily pain, general health perception, physical role functioning, emotional role functioning, social role functioning, and mental health [32]. The SF-36 has been validated for use with diverse age populations and diagnoses, with high construct and content validity and reliability of 0.90 [32].

## 3. Results

### 3.1. Data Preparation

#### 3.1.1. Analysis of Group Dropouts

A total of 36 groups were run between 2002 and 2011. Patients were considered to have completed the program if they attended 13 out of 20 scheduled sessions. Out of the 311 who entered the program, 214 (68.8%) completed the program (“completers”) and 97 (31.2%) dropped out (“noncompleters”), with the attrition rate ranging between 20 and 40%. The group size ranged from 7 to 11 people.

Completers and noncompleters were compared on demographic variables and baseline questionnaires using independent samples *t*-tests (see Table 1). The two groups did not differ with respect to years in pain or number of diagnoses. They did differ significantly in terms of age; completers were slightly older than noncompleters.

Chi-square tests were used to compare the completers and noncompleters on categorical variables. Pearson Chi-square 2 × 2 analysis using gender (male, female) and completion status (completer, noncompleter) did not show a significant relationship, *χ* ^2^ (1, *N* = 311) = 0.683, *p* = 0.409. Pearson Chi-square analysis using primary pain diagnosis (chronic widespread pain, neck and back pain, neuropathic pain, and arthritis) and completion status (completer, noncompleter) did not show a significant relationship, *χ* ^2^ (3, *N* = 283) = 5.85, *p* = 0.119.

The completers consisted of a total of 214 people. The number of sessions attended by completers ranged from 13 to 20 sessions (*M* = 16.57, SD = 2.25). Of those, 170 (79.4%) were women and 44 (20.6%) were men. The primary pain diagnoses of those who completed the program are presented in Table 2. Four categories of pain diagnosis accounted for the majority of the diagnoses (68.7%), as indicated in Table 2.

### 3.2. Changes over Treatment

The pre- and posttreatment questionnaire scores for the completers were compared using a paired samples *t*-test (alpha = 0.05, two-tailed). All of the questionnaires showed statistically significant changes in the direction of improved health. There were significant decreases in all of the SF-MPQ subscales. There were also significant decreases in scores for depression, anxiety, and pain disability. The Vanderbilt subscales showed significant decreases in passive coping and significant increases in active coping. The SF-36 showed significant increases on all of the subscales (see Table 3).

### 3.3. Number of Diagnoses

We examined the relationship between the posttreatment outcome variables and the number of diagnoses using Pearson correlation coefficients. The results did not show a statistically significant relationship between the number of diagnoses and the change in outcome variables (see Table 4).

### 3.4. Diagnostic Groups

We compared diagnostic group changes over time using a mixed design analysis of variance (ANOVA). In order to have sufficient statistical power, we restricted the analyses to the four most common diagnoses: chronic widespread pain, neck and back pain, neuropathic pain, and arthritis. The effects over time for these four specific groups are similar to those that were observed when all of the diagnostic groups were analysed together.

In summary, all of the outcome measures showed a statistically significant main effect of time in the direction of improved health except for SF-MPQ Physical Function and General Health subscales, which showed nonsignificant changes (see Tables 5 and 6). Only the SF-36 Energy subscale showed a significant main effect of group, although follow-up post hoc analysis using Tukey's HSD did not show any significant differences among diagnostic groups (*p*'s > 0.068). Analysis of the main effects of treatment and the treatment by group interaction did not show statistically significant differences.

### 3.5. Gender

Repeated measures ANOVA was used to examine systematic differences according to gender (male, female) for each outcome variable over time (pre- and post-PMG). Consistent with the above analyses, the main effect for time significantly improved from pretreatment to posttreatment for all variables with the exception of the SF-MPQ Sensory subscale. Only the Vanderbilt Active scale showed a significant main effect of gender, *F*(1, 169) = 5.01, *p* = 0.026, with women reporting higher levels of active coping (*M* = 3.19, SD = 0.05) than men (*M* = 2.94, SD = 0.10). No interaction effects were significant.

Correlations between age and years of pain were examined with the change in the outcome variables. The only correlation that was significant was between years of pain and the change in the SF-MPQ Affective scale, *r*(136) = −0.176, *p* = 0.041.

## 4. Discussion

The results of the present study show that PMGs can significantly reduce symptoms of pain, pain disability, depression, and anxiety. The significant goal for patients in this instance for these types of programs is to learn to better cope with the pain that they have on an ongoing basis. Pain management is significantly different from pain eradication. If such programs as the Bridgepoint PMG can assist clients to better live their lives, this will be considered as success in this context.

It is important that our analyses showed few differences between individuals that completed and that did not complete the PMG. This then allows us to conclude that our sample of 214 fairly accurately represents the entire study sample of 311. We confirmed this for their years of pain and their number of diagnoses, although the completers were a bit older than the noncompleters. However, the fact that there was no significant difference for either gender or primary diagnosis enables us to state that the positive outcomes found for the PMG program are likely to occur equally for male and female participants and regardless of the diagnosis that brought them to Bridgepoint. These are important findings given that this facility specializes in complex chronic conditions. If the number of diagnoses of individuals participating in the PMG is not relevant to their outcome, this confirms that the program is potentially equally helpful for them, regardless of the number of complex health conditions.

The results also indicate that the PMG can be helpful to the Bridgepoint outpatients even if they have experienced pain for a long time. This appears to contradict the commonly held assumption, both by individuals with chronic pain and also typically by many of their professional and family caregivers, that the pain “will never go away.” However, perhaps both perspectives may be correct. In most cases, PMG participants report in the final group session that their pain persisted to some extent and in varying degrees. However, they also report that the pain does not bother or hamper them as much after attending the Bridgepoint PMG sessions.

Future research should focus not only on the qualitative subjective perceptions of chronic pain as in this study, but also on its personal impact on the lives of these outpatients. Thus, the quantitative measurement of pain using pain perception instruments is clearly important, but tools such as the Active and Passive Coping Scale may in fact be even more important from a research methodology perspective in getting at the root issue of* managing* the pain, not necessarily seeking or expecting to eradicate the pain. This speaks to the basic approach used in CBT for chronic pain, and it appears to work well at Bridgepoint, at least for the time during which the patients attend 13 or more of the 20 sessions.

The larger issue is whether the positive effects of the self-management strategies taught in the PMG are sustained following completion of the program and if so, for how long. This raises the question of the retention of benefits gained from the PMG. We are planning to conduct a follow-up study in the future.

While the overall quantitative results of the study were positive, there is still the question of what the clinical implications of the PMG are and whether it produces positive results. In this respect, further research would benefit from qualitative analysis (e.g., small focus groups, key informant interviews) to examine whether the participants felt that they were better able to manage and actively cope with their pain after completing the PMG, since the PMG is not designed to “cure” their pain, but rather to teach techniques for better pain management. It is also notable that scores on depression and anxiety scales remained high even following a significant drop in reported symptoms after treatment (48%/58.5% of participants scored in the moderate to severe range for depression and anxiety, resp.). These results point to the intractability of the patients' pain problems notwithstanding the observed benefits of the PMG. Addressing the ongoing problems of depression and anxiety more directly in the group sessions may help to bring down these scores further.

This study has several limitations. Most notably, a control group that would provide further evidence of the effectiveness of the Bridgepoint PMGs was not included. However, the present data were collected in a “real-world” clinical setting rather than the context of a formally constructed randomized, controlled trial: this may provide more accurate insights into the advantages and disadvantages of conducting PMGs in the real world. Additionally, many of the completed questionnaires had missing data. Having staff ensure that all participants complete the pre- and posttest outcome measure questionnaires could help increase the sample size and reduce any missing data. An additional study limitation, which may somewhat reduce the generalizability of the results based on gender, is the fact that there were more female participants than male participants. While it would be challenging to ensure that there are relatively equal numbers of male and female participants in the groups, since it depends on waitlist referrals, it would be beneficial to have somewhat equal male and female participants in the groups. Furthermore, group dynamics are important for the group sessions. However, this is something that cannot be controlled for. In one instance, a lone male group member changed to a later group since he did not want to be the only male group member.

Future directions for the Bridgepoint PMG for a prospective study could be to have a randomized control/waitlist control group. There could also be all male groups, all female groups, and mixed gender groups to examine from a qualitative perspective how group dynamics play a part in the sessions. Future research should include more follow-up analyses following completion of PMGs to see whether treatment gains obtained during the program are maintained over time.

## 5. Conclusions

The most significant changes occurred in mood (improved levels of depression and anxiety) and in a shift to employing active versus passive coping strategies. These results are consistent with previously reported benefits of CBT for chronic pain patients and in programs that combine CBT with physical exercise. While there was a decrease in pain levels, this was less noticeable. This is consistent with previously reported outcomes of other similar CBT pain management programs.

It is important to again note that the overall goal of such programs is not specifically to decrease pain. All of the clients must by definition and inclusion requirements have had chronic pain, that is, pain lasting 6 months or more. The clients have all tried numerous approaches to decrease their pain which have not worked. Thus, this group of patients with intractable pain have a very poor prognosis for elimination of their pain. On the other hand, our analysis reinforces the sense that learning skills to manage pain can improve the mood and QOL and can even reduce pain levels in these patients.

## Figures and Tables

**Table 1:** Characteristics of individuals that completed the pain management group program (“completers”; *N* = 214) in comparison to those that did not complete it (“noncompleters”; *N* = 97).

Variable	Group		*t*	df	*p*	*d*
	Completers *M* (SD)	Noncompleters *M* (SD)				
Years of pain	9.1 (10.8)	10.0 (10.4)	0.695	305	0.488	0.084
Age	53.5 (12.0)	50.0 (13.0)	2.306	309	0.022^*∗*^	0.280
Number of diagnoses	2.9 (1.4)	2.9 (1.4)	0.298	309	0.766	0

^*∗*^Significant at *p* ≤ 0.05.

**Table 2:** Primary pain diagnoses of study participants.

Primary pain diagnosis	Frequency	Percentage
Chronic widespread pain	71	33.2
Neck and back pain	60	28.0
Arthritis	38	17.8
Neuropathic pain	27	12.6
Headache	10	4.7
Other	8	3.7

**Table 3:** Questionnaire scores before and after treatment in a pain management group.

Questionnaire	Pretreatment	Posttreatment	*t*	df	*p*	*d* ^*∗*^
	*M* (SD)	*M* (SD)				
BDI-II	27.2 (14.0)	21.8 (13.9)	6.5	127	<0.0005	0.567
BAI	25.2 (14.4)	21.5 (14.7)	3.7	122	<0.0005	0.335
SF-MPQ						
Sensory	19.3 (7.5)	17.9 (6.7)	2.200	104	0.030	0.226
Affective	7.0 (3.3)	6.2 (3.4)	3.163	136	0.002	0.261
Total	26.3 (10.0)	24.1 (9.6)	2.652	100	0.009	0.262
VAS	6.7 (2.2)	6.1 (2.3)	3.160	148	0.002	0.268
PDI	47.0 (13.8)	43.2 (13.6)	4.1	130	<0.0005	0.355
VPMI						
Active	3.0 (0.7)	3.3 (0.7)	−5.148	170	<0.0005	−0.449
Passive	3.4 (0.7)	3.2 (0.7)	6.198	173	<0.0005	0.373
SF-36						
Physical function	31.0 (21.6)	34.4 (22.0)	−2.312	179	0.022	−0.175
Role limit: physical	5.9 (16.6)	13.1 (26.3)	−3.897	176	<0.0005	−0.310
Role limit: emotional	24.9 (38.6)	35.2 (41.5)	−3.281	177	0.001	−0.247
Energy	28.5 (19.2)	33.2 (19.0)	−3.793	173	<0.0005	−0.286
Emotional wellbeing	48.1 (22.8)	55.9 (22.1)	−6.516	174	<0.0005	−0.495
Social	35.2 (26.4)	43.9 (26.7)	−4.912	180	<0.0005	−0.365
Pain	23.2 (16.0)	30.6 (18.6)	−5.712	174	<0.0005	−0.439
General health	35.0 (19.1)	37.2 (22.2)	−1.997	181	0.047	−0.149

*Note*. BDI-II: Beck Depression Inventory; BAI: Beck Anxiety Inventory; VAS: visual analogue scale; PDI: Pain Disability Index; VPMI: Vanderbilt Pain Management Inventory; SF-36: Short-Form Health Survey.

^*∗*^Corrected for dependence between means [10].

**Table 4:** Correlations between questionnaire outcome variables posttreatment and number of reported diagnoses of study participants.

Questionnaire	df	*r*	*p*
BDI-II	128	−0.001	0.995
BAI	123	−0.069	0.445
SF-MPQ			
Sensory	105	−0.008	0.938
Affective	137	−0.080	0.353
Total	101	−0.045	0.655
PDI	131	0.069	0.437
VPMI			
Active	171	−0.033	0.672
Passive	174	0.008	0.913
SF-36			
Physical function	180	0.030	0.691
Role limit: physical	177	0.009	0.902
Role limit: emotional	178	0.055	0.470
Energy	174	0.005	0.952
Emotional wellbeing	175	−0.021	0.784
Social	181	−0.035	0.640
Pain	175	0.004	0.957
General health	182	−0.015	0.840

*Note*. BDI-II: Beck Depression Inventory; BAI: Beck Anxiety Inventory; VAS: visual analogue scale; PDI: Pain Disability Index; VPMI: Vanderbilt Pain Management Inventory; SF-36: Short-Form Health Survey.

**Table 5:** Pre- and posttreatment scores for outcome variables according to diagnostic group.

	Chronic widespread pain		Neck and back pain		Neuropathic pain		Arthritis	
	Pre	Post	Pre	Post	Pre	Post	Pre	Post
	*M* (SD)	*M* (SD)	*M* (SD)	*M* (SD)	*M* (SD)	*M* (SD)	*M* (SD)	*M* (SD)
BDI-II	28.54 (13.82)	23.61 (14.82)	26.74 (13.89)	20.86 (13.35)	24.33 (12.76)	17.81 (11.23)	28.11 (12.85)	21.89 (12.20)
BAI	26.67 (16.06)	24.83 (13.48)	24.74 (13.76)	20.40 (15.66)	22.06 (9.01)	16.63 (11.06)	25.59 (15.17)	17.78 (11.89)
SF-MPQ								
Sensory	19.42 (6.99)	17.79 (5.94)	17.67 (8.08)	17.40 (5.94)	19.41 (8.36)	14.64 (6.73)	21.66 (7.61)	22.09 (5.40)
Affective	6.73 (3.66)	6.42 (3.65)	6.74 (2.94)	6.14 (3.07)	7.32 (2.94)	5.68 (4.07)	7.03 (3.13)	6.78 (2.94)
Total	26.21 (9.73)	24.47 (8.87)	24.71 (10.67)	23.98 (10.78)	26.77 (11.27)	20.09 (7.54)	28.40 (10.25)	28.83 (7.54)
PDI	48.26 (11.88)	45.77 (10.08)	47.55 (14.27)	43.47 (15.49)	45.00 (16.40)	40.50 (12.49)	47.97 (12.82)	44.85 (13.92)
VPMI								
Active	2.88 (0.77)	3.21 (0.69)	3.00 (0.67)	3.29 (0.63)	3.15 (0.67)	3.25 (0.59)	3.11 (0.70)	3.21 (0.59)
Passive	3.49 (0.65)	3.25 (0.63)	3.37 (0.77)	3.13 (0.72)	3.31 (0.71)	3.01 (0.71)	3.39 (0.54)	3.05 (0.54)
SF-36								
Physical function	28.38 (19.81)	32.87 (22.46)	31.07 (20.84)	34.93 (23.05)	39.69 (23.03)	41.61 (20.51)	24.61 (21.52)	26.13 (18.58)
Role limit: physical	3.57 (12.34)	6.43 (12.64)	8.80 (16.93)	10.19 (20.90)	4.17 (20.41)	21.88 (34.03)	4.76 (16.99)	19.05 (36.15)
Role limit: emotional	32.41 (42.53)	32.41 (41.01)	24.85 (39.14)	33.33 (41.45)	22.22 (37.64)	38.89 (41.31)	12.70 (26.82)	34.92 (45.31)
Energy	23.33 (17.49)	24.49 (17.76)	31.20 (20.20)	35.44 (18.94)	33.19 (18.96)	38.48 (17.67)	27.14 (17.36)	37.54 (17.60)
Emotional wellbeing	46.60 (22.89)	53.22 (24.51)	52.15 (22.14)	59.54 (19.71)	48.17 (20.95)	57.67 (21.00)	45.90 (22.29)	57.33 (20.76)
Social	33.68 (26.19)	37.85 (27.95)	39.06 (26.44)	48.88 (27.83)	33.85 (27.21)	41.15 (22.87)	33.33 (28.60)	47.62 (26.99)
Pain	22.93 (12.40)	27.86 (16.54)	23.54 (17.25)	32.17 (19.54)	24.48 (15.20)	32.40 (16.64)	20.25 (18.49)	32.50 (19.70)
General health	35.52 (19.30)	36.96 (21.85)	37.63 (19.59)	40.36 (22.88)	33.54 (17.22)	36.37 (21.89)	35.00 (17.25)	37.80 (19.78)

*Note*. BDI-II: Beck Depression Inventory; BAI: Beck Anxiety Inventory; VAS: visual analogue scale; PDI: Pain Disability Index; VPMI: Vanderbilt Pain Management Inventory; SF-36: Short-Form Health Survey.

**Table 6:** Results of a series of 4 × 2 mixed design ANOVA using group (chronic widespread pain, neck and back pain, neuropathic pain, and arthritis) and time (pretreatment, posttreatment).

Questionnaire	df	Effect of treatment			Effect of group			Treatment by group interaction		
		*F*	*p*	*η*_*p*_^2^	*F*	*p*	*η*_*p*_^2^	*F*	*p*	*η*_*p*_^2^
BDI-II	92	37.92	<0.001	0.292	0.39	0.758	0.013	0.40	0.757	0.013
BAI	87	14.48	<0.001	0.143	0.86	0.465	0.029	0.88	0.453	0.030
SF-MPQ										
Sensory	71	4.08	0.047	0.054	1.77	0.160	0.070	1.84	0.148	0.072
Affective	94	4.33	0.040	0.044	0.10	0.959	0.003	0.79	0.503	0.025
Total	67	3.97	0.050	0.056	0.94	0.428	0.040	1.58	0.202	0.066
PDI	93	8.78	0.004	0.086	0.45	0.721	0.014	0.15	0.928	0.005
VPMI										
Active	126	9.77	0.002	0.072	0.36	0.782	0.008	0.88	0.456	0.020
Passive	126	27.61	<0.001	0.180	0.61	0.611	0.014	0.19	0.903	0.005
SF-36										
Physical function	133	2.63	0.108	0.019	2.52	0.061	0.054	0.15	0.927	0.003
Role limit: physical	130	17.50	<0.001	0.119	1.20	0.313	0.027	3.73	0.063	0.079
Role limit: emotional	132	9.93	0.002	0.070	0.29	0.826	0.008	1.58	0.197	0.035
Energy	129	13.78	<0.001	0.096	3.12	0.028	0.068	1.64	0.184	0.037
Emotional wellbeing	131	35.95	<0.001	0.215	0.66	0.577	0.015	0.51	0.675	0.012
Social	133	15.90	<0.001	0.107	0.97	0.407	0.021	0.85	0.467	0.019
Pain	128	28.91	<0.001	0.184	0.28	0.843	0.006	0.84	0.475	0.019
General health	134	3.10	0.080	0.023	0.32	0.809	0.007	0.07	0.976	0.002

*Note*. BDI-II: Beck Depression Inventory; BAI: Beck Anxiety Inventory; VAS: visual analogue scale; PDI = Pain Disability Index; VPMI: Vanderbilt Pain Management Inventory; SF-36: Short-Form Health Survey.

**Table 7:** Description of the Bridgepoint Pain Management Group program by session number.

Session number	Session agenda	Homework
1	Introduction Set individual program goals Questionnaires Relaxation: theory & practice Qi Gong	(i) Program goals (ii) Relaxation exercise daily

2	Review individual program goals Right & left brain function Complete questionnaires Pain diary Hydrotherapy or gym	(i) Relaxation exercise daily (ii) 3 min breathing exercise (iii) Pain diary

3	Review goals & pain diaries Definitions of pain Physiology of pain Pain theories Exercise: assess negative thoughts Qi Gong	(i) Assess negative thoughts

4	Review use of relaxation & breathing Introduction to collage Dynamic imagery Relaxation: practice Hydrotherapy or gym	(i) Relaxation exercise daily (ii) Imagery exercise 4x a day

5	Review coping exercise Video: accepting the pain, discussion Discovering the “silver lining” Intro to cognitive behavioural approach Negative self-talk Flare-up plan Qi Gong	(i) Positive experiences

6	Imagery as a pain management technique Relaxation: practice Hydrotherapy or gym	(i) Relaxation tapes daily (ii) Draw pain picture

7	Review positive experiences Demonstration of cognitive model Practical application of the model (group work on self-talk) Qi Gong	(i) Self-talk exercise

8	Development of individual pain imagery Relaxation: practice Hydrotherapy or gym	(i) Relaxation exercise daily (ii) Practice pain imagery

9	Stress and its relationship with pain Group work: self-talk exercises Qi Gong	(i) Self-talk exercise

10	Sexuality & pain: guest speaker: sex therapist Further work on pain imagery Relaxation: practice Hydrotherapy or gym	(i) Relaxation exercise daily (ii) Practice pain imagery (iii) Review program goals

11	Review program goals Role of medications in chronic pain Stress video Group work: self-talk exercises Qi Gong	(i) Reducing stress (ii) Prepare for collage to be completed in Session 12

12	Collage exercise: group reporting Medications (cont'd) Further work on pain imagery Relaxation: practice Hydrotherapy or gym	(i) Relaxation exercise daily (ii) Practice pain imagery

13	Guest speaker: former group member Strategies for improved sleep quality Review collage exercise Qi Gong	(i) Sleep strategies (ii) Self-talk exercise (iii) Reducing stress

14	Energy conservation (OT, Sandy Duncan) Further work on pain imagery Relaxation: practice Hydrotherapy or gym	(i) Relaxation & imagery (ii) Anger styles

15	Therapeutic benefits of humour (video) Anger management Review sleep strategies Discussion: self-talk/stressful situations Qi Gong	(i) Humour exercise (ii) Anger exercise

16	Review exercise: anger episode analysis Review humour exercise Planning for family session^1^ Relaxation: practice Hydrotherapy or gym	(i) Continue use of humour (ii) Relaxation & imagery

17	Family session^1^ Qi Gong	(i) Review/complete cognitive exercises

18	Review of family session Nutrition and pain (nursing) Introduction to mindfulness meditation Hydrotherapy or gym	(i) Relaxation: practice

19	Discussion of community resources ACPA video “getting involved” Final review cognitive strategies Discussion of other related pain topics Qi Gong	(i) Review initial goals (ii) Prepare future goals

20	Review individual goals/set future goals Program evaluation Graduation: congratulations! Book follow-up session Hydrotherapy or gym	(i) Apply strategies learned in 10-week program

*Note*. Group discussion sessions are 2 hours in length, followed by group exercise for 30–60 minutes.

^1^During the program, one session is arranged for family members and friends of participants to attend in order to learn about chronic pain and how to support their loved ones. This is typically held on Session 17, but it could be moved to a different session if that would allow more people to attend.

## Appendix

See Table 7.

## References

[1] Merskey H., Bogduk H. (1994). *Classification of Chronic Pain: Descriptions of Chronic Pain Syndromes and Definitions of Pain Terms*.

[2] Epping-Jordan J. E., Wahlgren D. R., Williams R. A. (1998). Transition to chronic pain in men with low back pain: predictive relationships among pain intensity, disability, and depressive symptoms. *Health Psychology*.

[3] Ravens-Sieberer U., Bullinger M. (1998). Assessing health-related quality of life in chronically ill children with the German KINDL: first psychometric and content analytical results. *Quality of Life Research*.

[4] Boulanger A., Clark A. J., Squire P., Cui E., Horbay G. L. A. (2007). Chronic pain in Canada: have we improved our management of chronic noncancer pain?. *Pain Research & Management*.

[5] Turk D. C., Swanson K. S., Tunks E. R. (2008). Psychological approaches in the treatment of chronic pain patients—when pills, scalpels, and needles are not enough. *Canadian Journal of Psychiatry*.

[6] Kuluski K., Hoang S. N., Schaink A. K. (2013). The care delivery experience of hospitalized patients with complex chronic disease. *Health Expectations*.

[7] Moulin D. E., Clark A. J., Speechley M., Morley-Forster P. K. (2002). Chronic pain in Canad—prevalence, treatment, impact and the role of opioid analgesia. *Pain Research and Management*.

[8] Loeser J. D. (1999). Economic implications of pain management. *Acta Anaesthesiologica Scandinavica*.

[9] Smith B. H., Elliott A. M., Hannaford P. C. (2004). Is chronic pain a distinct diagnosis in primary care? Evidence arising from the Royal College of General Practitioners' Oral Contraception study. *Family Practice*.

[10] Morris S. B., DeShon R. P. (2002). Combining effect size estimates in meta-analysis with repeated measures and independent-groups designs. *Psychological Methods*.

[11] Geisser M. E., Roth R. S., Theisen M. E., Robinson M. E., Riley J. L. (2000). Negative affect, self-report of depressive symptoms, and clinical depression: relation to the experience of chronic pain. *Clinical Journal of Pain*.

[12] Strong J. (1998). Incorporating cognitive-behavioral therapy with occupational therapy: a comparative study with patients with low back pain. *Journal of Occupational Rehabilitation*.

[13] Nielson W. R., Jensen M. P. (2004). Relationship between changes in coping and treatment outcome in patients with Fibromyalgia Syndrome. *Pain*.

[14] Dysvik E., Kvaløy J. T., Stokkeland R., Natvig G. K. (2010). The effectiveness of a multidisciplinary pain management programme managing chronic pain on pain perceptions, health-related quality of life and stages of change-a non-randomized controlled study. *International Journal of Nursing Studies*.

[15] Winterowd C., Beck A. T., Gruener D. (2003). *Cognitive Therapy with Chronic Pain Patients*.

[16] Taylor S. E., Sirois F. M. (2012). *Health Psychology (Second Canadian Edition)*.

[17] McCracken L. M., Turk D. C. (2002). Behavioral and cognitive–behavioral treatment for chronic pain: outcome, predictors of outcome, and treatment process. *Spine*.

[18] Dysvik E., Guttormsen Vinsnes A., Eikeland O.-J. (2004). The effectiveness of a multidisciplinary pain management programme managing chronic pain. *International Journal of Nursing Practice*.

[19] Redondo J. R., Justo C. M., Moraleda F. V. (2004). Long-term efficacy of therapy in patients with fibromyalgia: a physical exercise-based program and a cognitive-behavioral approach. *Arthritis Care & Research*.

[20] Disorbio J. M., Bruns D., Barolat G. (2006). Assessment and treatment of chronic pain. *Practical Pain Management*.

[21] Turk D. C., Okifuji A. (2002). Psychological factors in chronic pain: evolution and revolution. *Journal of Consulting and Clinical Psychology*.

[22] Joos B., Uebelhart D., Michel B. A., Sprott H. (2004). Influence of an outpatient multidisciplinary pain management program on the health-related quality of life and the physical fitness of chronic pain patients. *Journal of Negative Results in BioMedicine*.

[23] Qigong Institute http://www.qigonginstitute.org/category/4/getting-started.

[24] Melzack R. (1987). The short-form McGill pain questionnaire. *Pain*.

[25] Grafton K. V., Foster N. E., Wright C. C. (2005). Test-retest reliability of the Short-Form McGill Pain Questionnaire: assessment of intraclass correlation coefficients and limits of agreement in patients with osteoarthritis. *The Clinical Journal of Pain*.

[26] Melzack R., Katz J., McMahon S., Koltzenburg M., Tracey I., Turk D. C. (2013). Pain measurement in adult patients. *Wall & Melzack's Textbook of Pain*.

[27] Beck A. T., Steer R. A., Brown G. K. (1996). *Beck Depression Inventory-II*.

[28] Beck A. (2000). *Beck InterpreTrak. Computer Software*.

[29] Beck A. T., Steer R. A.

[30] Tait R. C., Chibnall J. T., Krause S. (1990). The Pain Disability Index: psychometric properties. *Pain*.

[31] Brown G. K., Nicassio P. M. (1987). Development of a questionnaire for the assessment of active and passive coping strategies in chronic pain patients. *Pain*.

[32] Ware J. E. (2000). SF-36 Health Survey update. *Spine*.

